# Enhancing bone regeneration: Unleashing the potential of magnetic nanoparticles in a microtissue model

**DOI:** 10.1111/jcmm.70040

**Published:** 2024-09-01

**Authors:** Maryam Dousti, Shima Parsa, Farnaz Sani, Elham Bagherzadeh, Zahra Zamanzadeh, Mahintaj Dara, Mahsa Sani, Negar Azarpira

**Affiliations:** ^1^ Shiraz Institute for Stem Cell and Regenerative Medicine Shiraz University of Medical Science Shiraz Iran; ^2^ Department of Genetics, Faculty of Biological Sciences and Technology Shahid Ashrafi Esfahani University Isfahan Iran; ^3^ R and D Manager, Preimure BV Utrecht The Netherlands; ^4^ Stem Cells Technology Research Center Shiraz University of Medical Sciences Shiraz Iran; ^5^ Tissue Engineering Department, School of Advanced Medical Science and Technology Shiraz University of Medical Science Shiraz Iran; ^6^ Transplant Research Center Shiraz University of Medical Science Shiraz Iran

**Keywords:** angiogenesis, Fe_3_O_4_, magnetic nanoparticle, microtissue, osteogenesis

## Abstract

Bone tissue engineering addresses the limitations of autologous resources and the risk of allograft disease transmission in bone diseases. In this regard, engineered three‐dimensional (3D) models emerge as biomimetic alternatives to natural tissues, replicating intracellular communication. Moreover, the unique properties of super‐paramagnetic iron oxide nanoparticles (SPIONs) were shown to promote bone regeneration via enhanced osteogenesis and angiogenesis in bone models. This study aimed to investigate the effects of SPION on both osteogenesis and angiogenesis and characterized a co‐culture of Human umbilical vein endothelial cells (HUVEC) and MG‐63 cells as a model of bone microtissue. HUVECs: MG‐63s with a ratio of 4:1 demonstrated the best results among other cell ratios, and 50 μg/mL of SPION was the optimum concentration for maximum survival, cell migration and mineralization. In addition, the data from gene expression illustrated that the expression of osteogenesis‐related genes, including osteopontin, osteocalcin, alkaline phosphatase, and collagen‐I, as well as the expression of the angiogenesis‐related marker, CD‐31, and the tube formation, is significantly elevated when the 50 μg/mL concentration of SPION is applied to the microtissue samples. SPION application in a designed 3D bone microtissue model involving a co‐culture of osteoblast and endothelial cells resulted in increased expression of specific markers related to angiogenesis and osteogenesis. This includes the design of a novel biomimetic model to boost blood compatibility and biocompatibility of primary materials while promoting osteogenic activity in microtissue bone models. Moreover, this can improve interaction with surrounding tissues and broaden the knowledge to promote superior‐performance implants, preventing device failure.

## 1 INTRODUCTION

Significant bone defects caused by trauma, tumours and infection are generally considered critical clinical challenges as they cause a delayed union or nonunion in bones. This can compromise musculoskeletal function, leaving surgical techniques such as autografting the only available option.^1^, ^2^ However, autografting is restricted due to substantial drawbacks such as limited graft supply, donor complications and disease transmission. In this regard, bone tissue engineering has been demonstrated to be promising in providing autograft alternatives such as in‐vitro bone scaffolds as well as tissue‐engineered microtissues mimicking the body's natural systems.^1^ Significantly, three‐dimensional (3D) cell‐culture systems are eagerly anticipated to remodel the physiological environment, improving the diffusion and adhesion of essential elements, including proteins, growth factors and enzymes. This, in turn, ensures cell viability and promises the model's function.^3^

Despite significant advancements in this area over the past 20 years, bone tissue engineering techniques have yet to be used in clinical settings. This is primarily because of a lack of blood flow to the implanted location as well as the engineered tissue construct.^4^ Blood vessel development (angiogenesis) is crucial for osteogenesis, the process of bone generation, as the osteoblasts can't survive, and the process of bone repair will be stopped without an appropriate blood supply.^1^, ^5^ Hence, simulating the physiological bone tissue environment to create optimal conditions for developing new micro‐vessels within bone structures would greatly assist the issue. In this context, endothelial cells and osteoblasts interacting in a co‐culture setting to model an in‐vitro bone microtissue may bring promising outcomes.^6^

The complex anatomy of bone tissue has made regenerating this organ more complicated. For example, bone tissue has special mechanical characteristics that make it challenging for tissue engineering.^7^, ^8^ Recent studies have shown how the unique properties of super‐paramagnetic iron oxide nanoparticles (SPIONs) would be applicable to support bone tissue regeneration. Super‐paramagnetic iron oxide agents have previously been used as a magnetic resonance contrast in clinical trials. Ferumoxytol, a super‐paramagnetic iron oxide family member, has been approved for treating adult patients with chronic renal disease who are iron‐deficient.^9^ Its property in combating iron deficiency is due to its capability to contribute to iron homeostasis by being digested by ionization into iron ions.^10^, ^11^

In a bone tissue regenerating investigation, Fe_3_O_4_ nanoparticles (as a type of SPIONs) have been illustrated to promote the nanocomposite scaffold strength, calcium deposition and alkaline phosphatase (ALP) activity. Mechanotransduction is the most likely process to promote bone regeneration, which is the translation of continual mild magnetic forces applied on a cell to internal biochemical signals.^12^, ^13^, ^14^, ^15^ A static magnetic field (SMF) improved cartilage extracellular matrix and chondrogenesis, alleviating osteoarthritis in mice models. Moreover, it stimulated endogenous stem cell migration by triggering the Piezo1‐mediated SDF‐1/CXCR4 regulatory axis.^16^ In mouse models with type 1 diabetes, an SMF balanced the activity of bone cells with the management of iron metabolism and redox state, resulting in a higher bone quality.^17^ SMFs have also illustrated increased osteogenic differentiation via Smad4 higher expression.^18^ Electrodynamic interactions, magneto‐mechanical interactions and influences on electronic spin states are possible physical processes of SMF and its physiological consequences.^1^ Moreover, in an in‐vivo study, the histological examination showed increased blood vessel generation within bone development after a SPION‐loaded gelatin sponge administration.^19^ SPION could foster angiogenesis in the presence or absence of an SMF.^20^, ^21^, ^22^

The present study, for the first time, aimed to develop a microtissue involving Human umbilical vein endothelial cells (HUVECs) (representing the endothelial cells) and MG‐63s (representing the osteoblasts) cell lines in combination with Fe_3_O_4_ nanoparticles. This was to experiment with the osteogenesis and angiogenesis characteristics within this engineered bone microtissue. This kind of model would apply to comprehensive investigations and drug screening.

## 2 METHODS

### 2.1 Synthesis and preparation of iron oxide nanoparticles

The synthesis of nanoparticles (NPs) was carried out using the iron sources FeCl_2_·4H_2_O and FeCl_3_·6H_2_O (Merck), hydrochloric acid (HCl, 37%, Merck), deionized water (DI), and ammonia solution (NH_4_OH, 25% Merck) as the alkaline agent. The coprecipitation method was used to develop the SPIONs according to a procedure reported by Khodaei et al.^23^ Two distinct 2 M ferrous and 1 M ferric solutions were initially prepared in diluted HCl (2 M). Then, 1 mL of the ferrous solution is added to 4 mL of the ferric solution and stirred for homogenization. Afterward, 50 mL of 0.4 M ammonia solution was added drop‐wise with a rate of 0.75 mL/min under magnetic stirring. Finally, the obtained black precipitate is decanted and washed with DI water several times. Before cell biology assessments, the SPIONs were autoclaved and remained sterile in Dulbecco's Modified Eagle Medium/Nutrient Mixture F‐12 (DMEM/F12, Gibco, USA) supplied by Penicillin/Streptomycin 1% (Bioidea, Iran).

### 2.2 Characterization of nanoparticles

The crystalline structure of the nanoparticles was identified using x‐ray diffraction (XRD‐PW1800, Philips). The morphology of the nanoparticles was observed using a field emission scanning electron microscope (FE‐SEM; TESCAN, Brno, Czech Republic), and the average nanoparticle size was determined using a transmission electron microscope (TEM; FEI Tecnai 12; Philips). A vibrating‐sample magnetometer (VSM, Meghnatis Daghigh Kavir) was used to evaluate the magnetic properties of the synthesized nanoparticles.

### 2.3 Cell culture

The two cell types used in the study, HUVECs and osteoblast‐like cells (MG‐63s) were obtained from the Pasture Institute in Tehran, Iran. The cells were cultured routinely in DMEM/F12 medium (Gibco, USA) supplemented with 1× Glutamax (Bioidea, Iran), Penicillin–Streptomycin (100 U/mL) (Bioidea, Iran) and 10% (vol/vol) fetal bovine serum (FBS) (Gibco, USA). The cell cultures were maintained in a humidified atmosphere of 5% CO_2_ at 37°C. The cells were prepared for the experiments by trypsinizing them with 0.05% trypsin/EDTA (Shellmax, China) when they reached approximately 70%–80% confluency.

### 2.4 Microtissues preparation

HUVECs and MG‐63s were used to create bone microtissues in five different ratios: (1) HUVECs, (2) HUVECs/MG‐63s (4:1), (3) HUVECs/MG‐63 (2:1), (4) HUVECs/MG‐63s (3:2) and (5) MG‐63s. A suspension containing 10,000 cells in DMEM media was added to each well from a 96‐well Flat bottom plate. Before cell seeding, the wells were coated with a melted autoclaved solution of 1% agarose‐medium (w/v) (100 μL/well) and cooled for 20 min. The cultured microtissues were examined under an optical microscope on the first, third and seventh days.^24^, ^25^

#### 2.4.1 Viability and mineralization assessment of the microtissue models

The viability of cells within all co‐cultures microtissue groups was evaluated using the LIVE/DEAD assay, a fluorescent dye‐based method. To stain both live and dead cells, 5 mg/mL of fluorescein diacetate (FDA) and 2 mg/mL of propidium iodide (PI, both Sigma) were re‐suspended in a culture media without FBS. The microtissues were then incubated at room temperature for 5 min without light. The samples were then examined using a fluorescence microscope (Olympus BX51).

The Alizarin Red staining method and analysis of ALP expression levels were employed to assess mineralization. The Alizarin Red staining process fixed all the groups in 70% ethanol for 20 min, followed by rinsing with PBS to remove any excess fixative. Afterward, the fixed groups were subjected to an Alizarin Red stain for 30 min. Alizarin Red is a dye that selectively binds to calcium ions, allowing for the visualization of mineralized deposits. To quantify the extent of mineralization, the alizarin red stained cultures were incubated with 100 mM cetylpyridinium chloride, and optical density (OD) was measured using a spectrophotometer at a wavelength of 405 nm.

Alkaline phosphatase (ALP) activity was assessed using an ALP assay kit (Biorexfars, UK) following the instructions provided by the manufacturer. Diethanolamine Magnesium Chloride Preservative and P‐Nitrophenyl phosphate were mixed at 75%:25% and 1000 μL was added to 20 μL of the samples. Afterward, the ALP levels were quantified by spectrophotometer at 405 nm.

### 2.5 Determining MG‐63 cells proliferation and mineralization upon SPION treatment

Four categories of 2D MG‐63 cells treated with SPIONs were developed. SPION‐free MG‐63 cells were determined as the control group, and treated groups were exposed to 100, 50 and 25 μg/mL concentrations of SPIONs. Selecting the lower dosage range was based on previous reports illustrating cell toxicity of the higher doses of SPIONs.^26^ The experiments were applied on culture days three, five and seven.

To investigate the cell proliferation and toxicity of SPIONs in the different treatment groups, the 3‐(4,5‐dimethyl‐2‐thiazolyl)‐2,5‐diphenyl tetrazolium bromide (MTT) test (M5655, Sigma‐Aldrich) was employed. Each well received 200 μL of a 0.5 mg/mL MTT solution, which was then incubated at 37°C for 4 h. Subsequently, 100 μL of dimethyl sulfoxide (Merck) replaced the first solution. Afterward, the ODs were measured with a plate reader spectrometer (FLUOstar Omega®, BMG Labtech) at 570 nm wavelength. To evaluate the mineralization of MG‐63 cells after SPION administration, Alizarin Red S stain and APL levels were examined on days three, five and seven following the steps above.

### 2.6 Scratch wound and transwell assays

After determining the optimum ratio of the HUVECs/MG‐63s at 4:1, the scratch wound and transwell assays were performed to examine further the optimum SPION dosage treatment influencing cell migration and healing. A monolayer of 7 × 10^4^ cells containing HUVECs and MG‐63s (4:1) cultured in a 24‐well plate was scratched by a 200 μL pipette. The detached cells were washed out using PBS and observed using optical microscopy. Residual cells were then cultured in SPOIN‐free medium and different mediums containing SPOIN at 100, 50 and 25 μg/mL. After 24 h of incubation, the cells were evaluated using optical microscopy.

### 2.7 Tube formation assay

This experiment evaluated the angiogenesis influenced by co‐culturing HUVECs with MG‐63s and 50 μg/mL of SPION (based on the optimum dosage from the abovementioned observations). In this experiment, the co‐cultured HUVECs and MG‐63s at the ratio 4:1 treated with and without SPION (50 μg/mL) were compared to HUVECs alone. 1 × 10^5^ cells were cultured in a 24‐well plate coated with 2% neutralized COL‐I (Sivan Cells Company, Iran). The PKH26 Red Fluorescent Cell Linker Kit (Sigma, USA) was used as general cell membrane labeling of HUVECs according to the manufacturer's instructions to track the angiogenesis. Tube formations were evaluated using an inverted microscope after they were incubated at 37°C for 6 h. Additionally, cells were fixed in 4% paraformaldehyde (Sigma‐Aldrich, USA), and the nuclei of cells were stained with the blue fluorescent dye DAPI (4′,6‐diamidino‐2‐phenylindole). The tube formation was examined by fluorescence microscope (Olympus BX51).

### 2.8 Immunohistochemistry assessment

Immunohistochemistry (IHC) test was applied to observe CD31 expression as a marker of angiogenesis rate. HUVECs and MG‐63s co‐cultures at the ratio 4:1 treated with and without SPION (50 μg/mL), compared with HUVECs alone in the IHC test. In this regard, spheroids were washed using PBS after being fixed in 4% paraformaldehyde. Anti‐CD31 antibody (dilution 1:50; Abcam) was mixed in all groups and incubated overnight at 4°C. The samples were subjected to a secondary antibody treatment the next day (dilution 1:200; Abcam). Then, the DAB + chromogen substrate system (dilution 1:50; Dako) and HRP (dilution 1:10,000; Abcam) were employed for its identification. A light microscope was used to identify it.^27^

### 2.9 Gene expression analysis

Quantitative real‐time polymerase chain reaction (qRT‐PCR) was carried out to quantify the mRNA levels of collagen type I (COL1A1, NM_000088), osteocalcin (OCT, NM_199173), osteopontin (OPN, NM_000582), (ALP, NM_000478.6), β‐actin (ACTB, NM_001101) and cluster of differentiation 31 (CD31, NM_000442.5). Reverse transcription and PCR amplification were used with the precise primers for each target gene. Real‐time PCR equipment was used to measure the mRNA levels, and the cycle threshold values were used to conduct the analysis. An RNeasy Plus Mini Kit (QIAGEN) extracted the total RNA from the 3D models. The manufacturer's instructions used RevertAid H Minus First strand cDNA synthesis kit (Thermo Scientific, USA) to generate complementary DNA (cDNA). The cDNA allocations were kept at −20°C until further examination. Real‐time reverse transcription polymerase chain reaction (RT‐PCR) was carried out utilizing an Applied Biosystems StepOnePlusTM System (ABI, USA) and the SYBR® Premix Ex TaqTM II kit (Takara, Japan) to evaluate the relative gene expression. The reference gene B‐ACTINE was utilized for standardization. Additionally, the 2‐ΔΔCt technique was applied to calculate each gene's fold change in expression.

### 2.10 Data analysis

To collect all the data, a minimum of three repeats were employed. Error bars are used to illustrate standard deviation. Data were investigated by one‐way analysis of variance (ANOVA) and Tukey's post hoc multiple comparison test through GraphPad Prism software (version 8.0.1; CA, USA). Statistical significance was defined as a *p*‐value of 0.05 or less.

## 3 RESULTS

### 3.1 Characterization of nanoparticle

**FIGURE 1:**
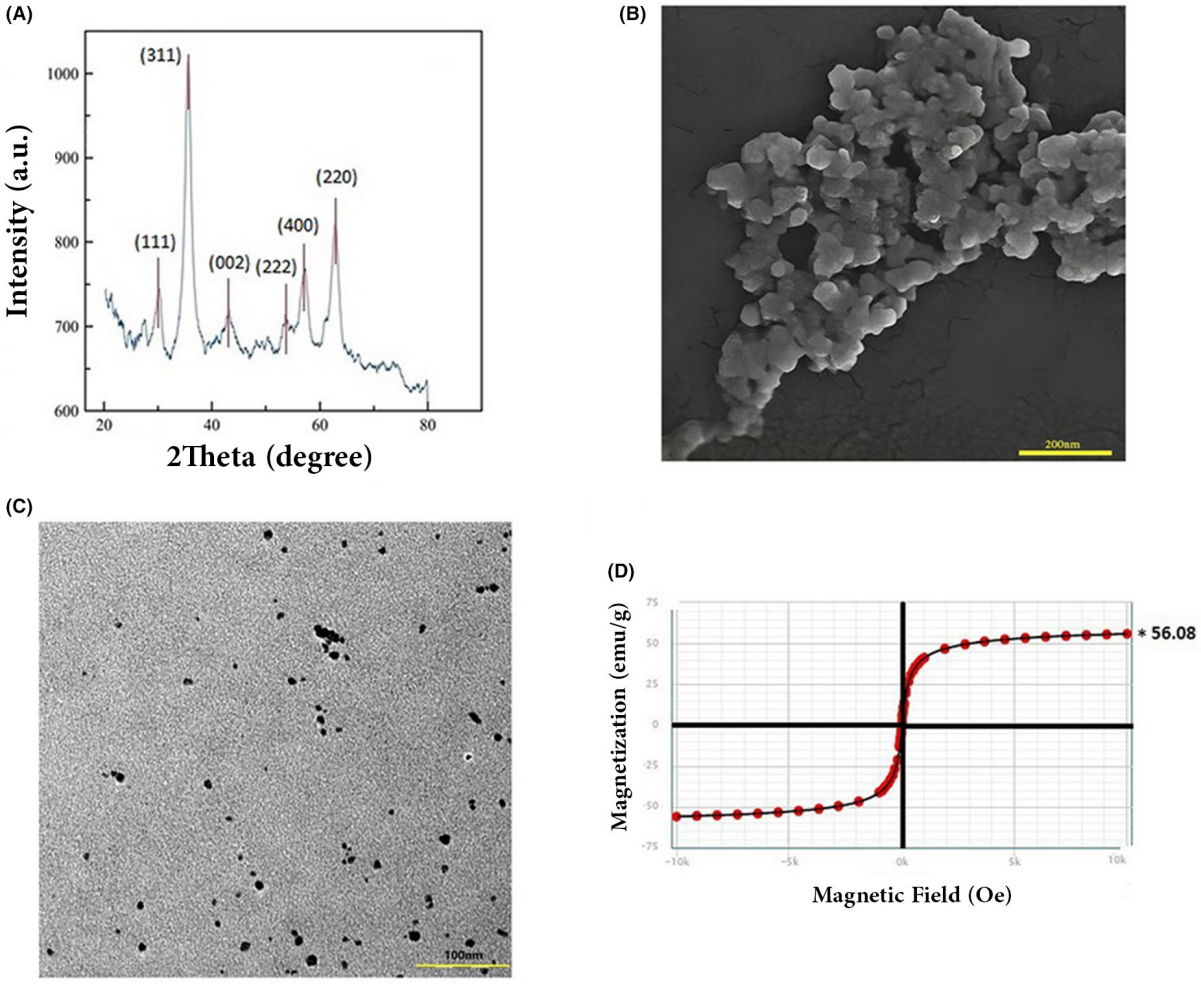
Nanoparticle characterization; XRD pattern of magnetite nanoparticles (A). SEM analysis of Fe_3_O_4_ (scale bar: 200 nm) (B). TEM images of Fe_3_O_4_ (scale bar: 100 nm) (C). VSM curvature of Fe_3_O_4_ nanoparticles (D).

The x‐ray diffraction pattern of the synthesized particles is illustrated in Figure 1A. Six major peaks can be distinguished at 20° of 30.06°, 35.57°, 43.0°, 53.77°, 57.05°, 62.77° and Coinciding with (111), (311), (002), (222), (400) and (220) crystalline planes, which are consistent with the diffraction pattern of Fe_3_O_4_ according to the ICDD 96‐900‐5842 database. Morphological evaluation of the synthesized Fe_3_O_4_ precipitate using FE‐SEM (Figure 1B) and TEM (Figure 1C) images shows that the particles were almost spherical and had a relatively uniform size distribution. TEM images were further analysed with Image J software to determine the average nanoparticle diameter in the range of 5–7 nm. Finally, the magnetization curve of the obtained nanoparticle showed that the nanoparticles were entirely super‐paramagnetic with a coercivity of close to zero Oe, and they have a saturation magnetization of 56.08 emu/g.

### 3.2 Development and viability of the microtissues models

**FIGURE 2:**
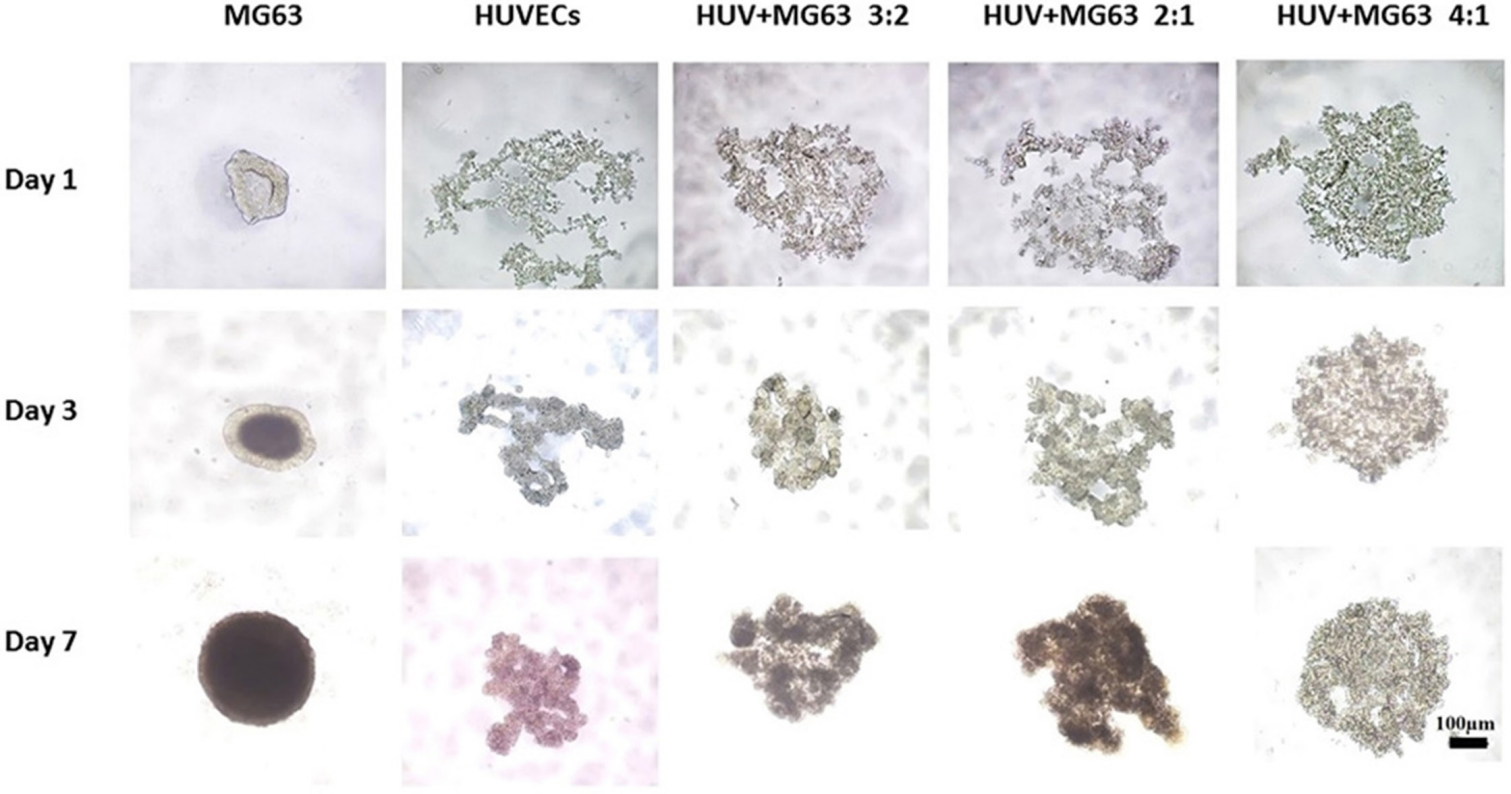
Microtissues development; Types of cell spheroids on Days 3, 1 and 7—MG‐63, HUVECs and HUVEC: MG‐63 groups with the ratios of 3:2, 2:1 and 4:1 (scale bar: 100 nm).

**FIGURE 3:**
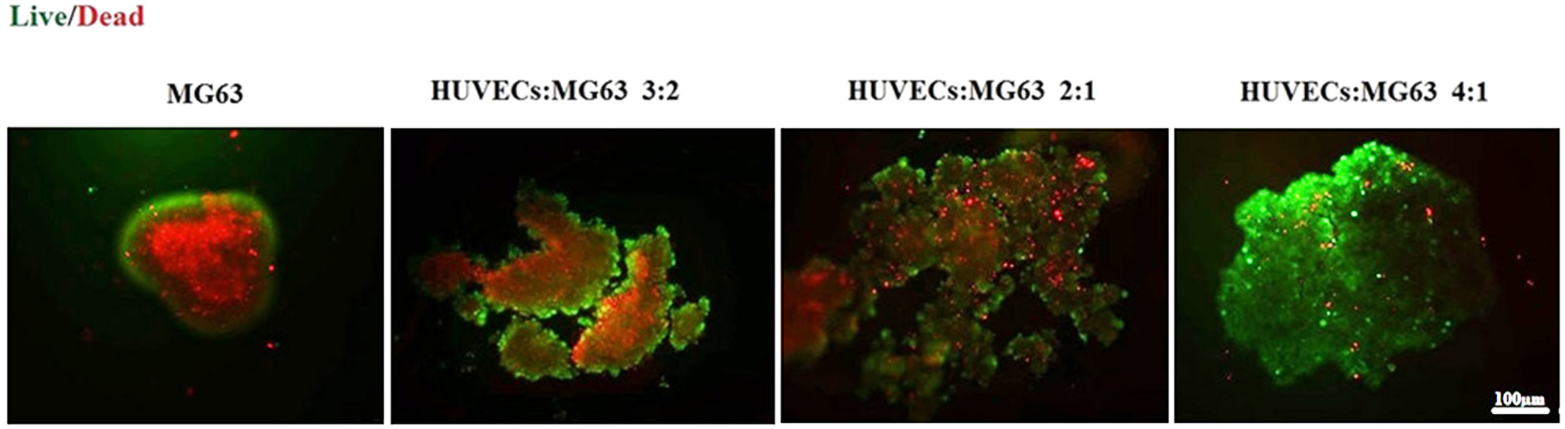
Microtissues viability; Live (green)/dead (red) assay to determine live and dead cells proportion within four different ratios: MG‐63s, HUVECs/MG‐63s (3:2), HUVECs/MG‐63s (2:1), and HUVECs/MG‐63s (4:1) (scale bars: 100 μm).

The bone microtissue models were established and characterized as previously described. Optical microscopy was employed to assess microtissue formation on Days 1, 3 and 7 of the culture (Figure 2). Based on the study using Image J, the spheroid's average diameter was less than 400 μm, which is the ideal size for a spheroid to benefit from not having a discernible necrotic core.^28^ On the third day of culture, the cells underwent self‐aggregation and the formation of compact microtissues was explicitly observed in the 4:1 HUVECs/MG‐63s ratio among the co‐cultured groups. To qualitatively verify the microtissues' viability in different proportions of HUVECs and MG‐63s, a LIVE/DEAD assay was applied. The MG‐63 spheroids did not demonstrate high viability, so it was hypothesized that microtissue with endothelial cells might show higher viability levels. The results confirmed the highest FDA expression in the models with a 4:1 ratio of HUVECs to MG‐63s (Figure 3).

### 3.3 Quantification of osteogenesis using various ratios of cells in the microtissue model

**FIGURE 4:**
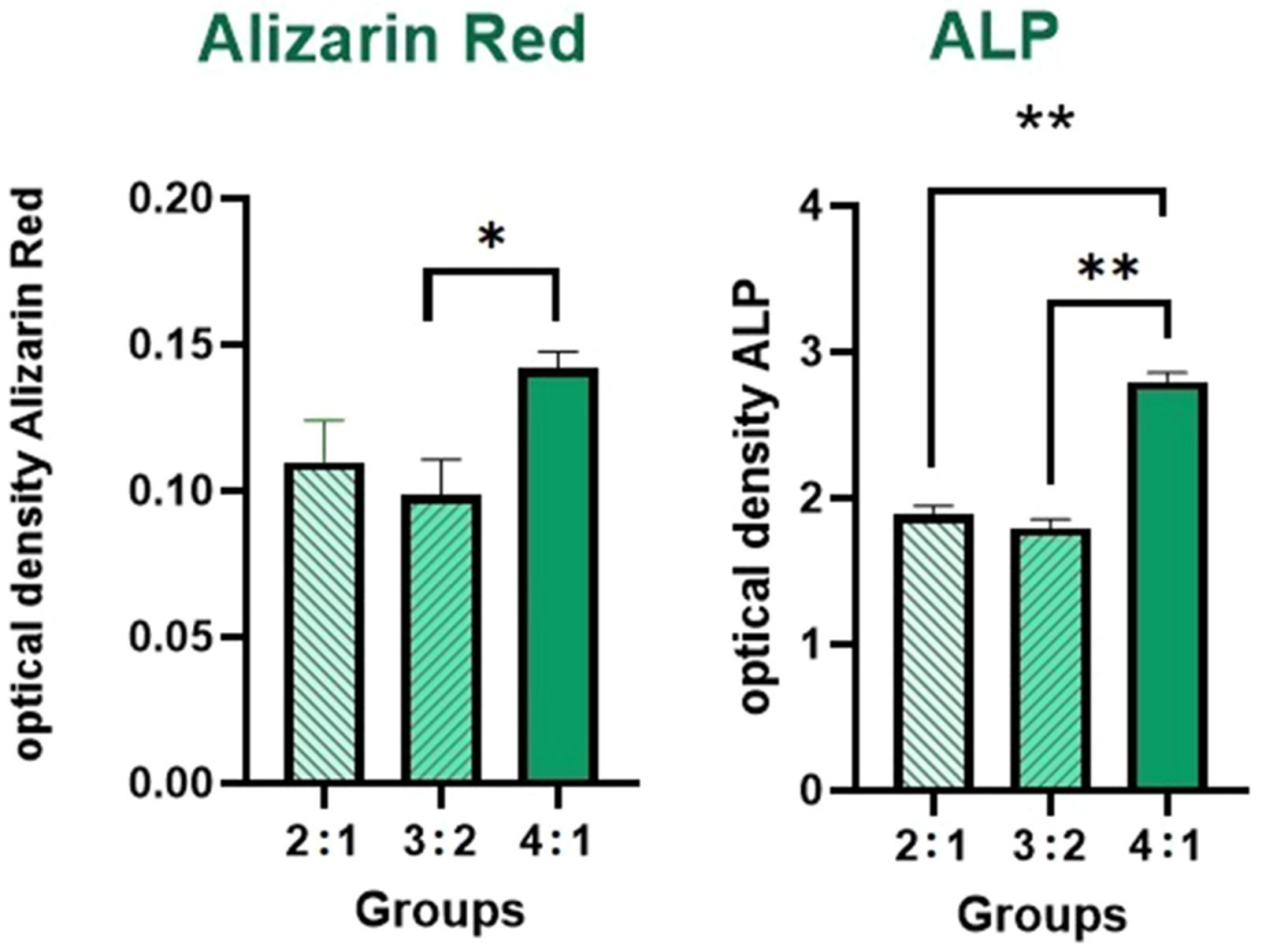
Osteogenesis quantification: Alizarin red and alkaline phosphatase (ALP) tests in three HUVEC: MG‐63 ratios showed the ratio of 4:1 has the highest levels of alizarin red and ALP secretion (*p < 0.05, **p < 0.01).

Alizarin Red reaction and ALP expression level tests were conducted to assess the osteogenesis levels quantitatively. The results of the Alizarin Red reaction demonstrated a higher mineralization/calcification when the ratio of HUVECs to MG‐63s was 4:1, and the difference was significant compared to HUVEC/MG‐63 (3:2) (*p*‐value = 0.0436) (Figure 4). These findings were further supported by the results of ALP expression levels, which showed a noticeable increase in the HUVEC/MG‐63 4:1 ratio group compared to other groups at ratios of HUVECs/MG‐63s (3:2) (*p*‐value = 0.0036) and HUVECs/MG‐63s (4:1) (*p*‐value = 0.0046) (Figure 4).

### 3.4 Optimal concentration of SPION administration enhanced cell proliferation and osteogenesis

**FIGURE 5:**
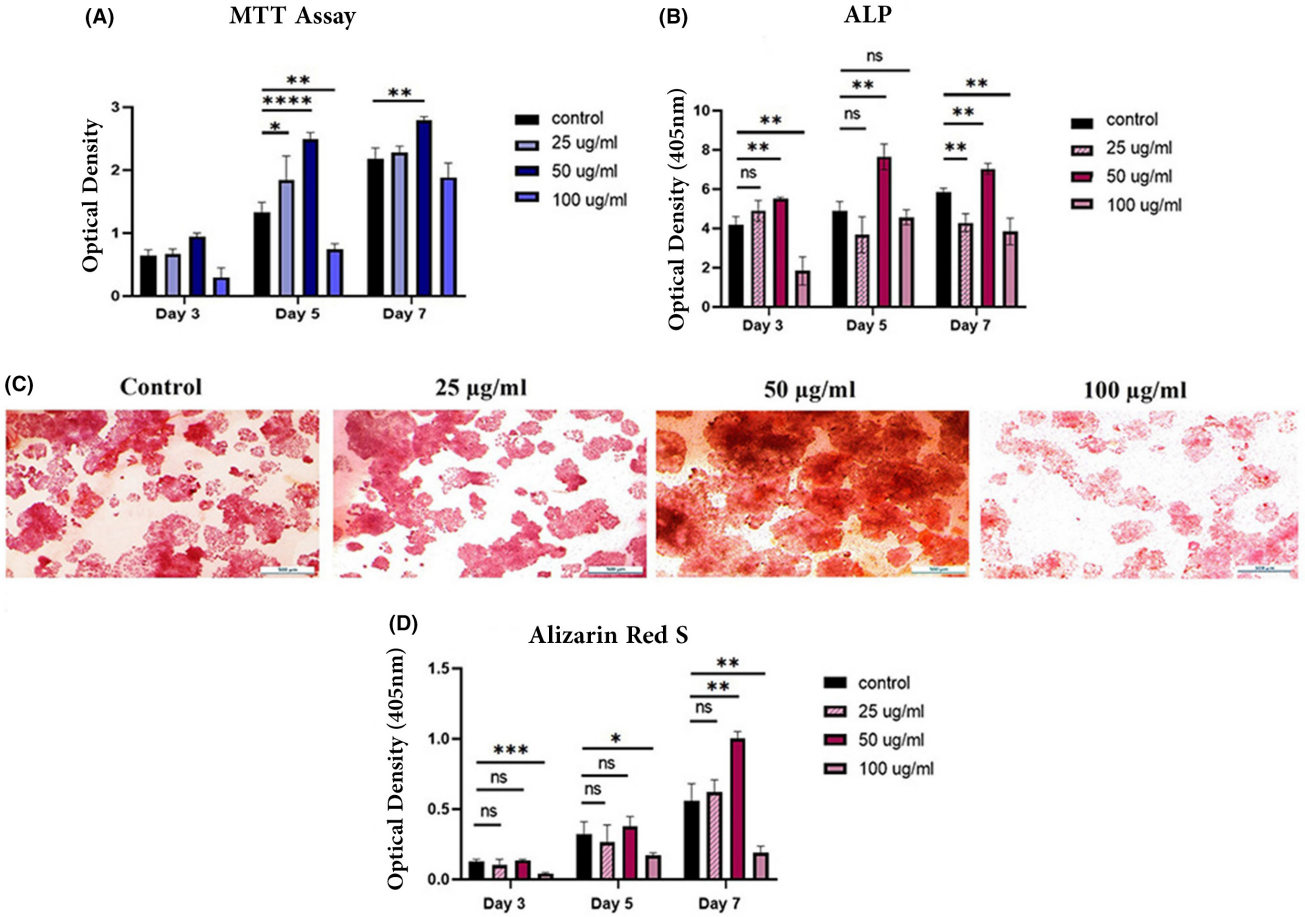
Cell proliferation and osteogenesis levels upon SPION administration on the 3rd, 5th and 7th culture days; MTT results for cell proliferation levels (A). ALP activity levels (B). Osteogenic differentiation levels stained by alizarin red (scale bars: 500 μm) (C), Alizarin Red assay to quantitatively determine osteogenic levels (D) (*p < 0.05, **p < 0.01, ***p < 0.001).

To investigate the effects of different concentrations of SPION on MG‐63s, microtissue cell proliferation (MTT assay) and their mineralization (Alizarin Red reaction and ALP expression assays) were evaluated upon SPION administration. SPION at concentrations of 100, 50 and 25 μg/mL were compared to a free‐SPION control group after 3, 5 and 7 days of treatment administration. Based on the MTT results, a concentration of 50 μg/mL of SPION exhibited a significant difference in cell proliferation compared to the control group on Day 5 (*p*‐value < 0.0001) and Day 7 (*p*‐value = 0.0069). In contrast, the 25 μg/mL concentration showed no noticeable difference compared to the control group on Days 3 and 7, while there was a significant difference on Day 5 (*p*‐value = 0.0363). However, the concentration of 100 μg/mL of SPION led to a decline in cell proliferation levels, which indicates its toxicity to the cells (Figure 5).

Administration of 50 μg/mL of SPION to MG‐63s demonstrated the highest levels of calcium deposition on the 7th day, indicating its potency in promoting mineralization. This observation was confirmed by the microscopic examination of the cells and their ODs at 405 nm in the Alizarin Red experiment (*p*‐value = 0.0069) (Figure 5). Additionally, the ALP expression test revealed that the osteoblasts exposed to 50 μg/mL of SPION exhibited significantly higher ALP expression levels compared to the control group on Day 3 (*p*‐value = 0.0064), Day 5 (*p*‐value = 0.0039) and Day 7 (*p*‐value = 0.0038) (Figure 5).

### 3.5 SPION increased cell viability in co‐cultured microtissue models

**FIGURE 6:**
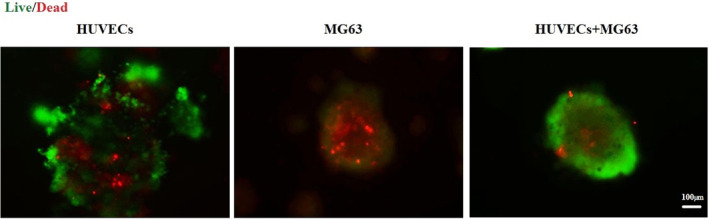
Live (green)/dead (red) test in spheroids exposed to 50 μg/mL of SPION; HUVEC: MG‐63 at a ratio of 4:1 has the highest viability compared to other groups (scale bars: 100 μm).

Three categories of the microtissues, including (1) HUVECs, (2) MG‐63s and (3) HUVECs/MG‐63s (4:1) were exposed to 50 μg/mL of SPION, and their viability was assessed using the Live/Dead experiment. The results indicated that the third group, HUVECs/MG‐63s (4:1), exhibited the highest viability levels (Figure 6). This suggests combining HUVECs and MG‐63s in a specific ratio enhances cell viability in 50 μg/mL of SPION.

### 3.6 SPION enhanced cells migrations and tubular formation

**FIGURE 7:**
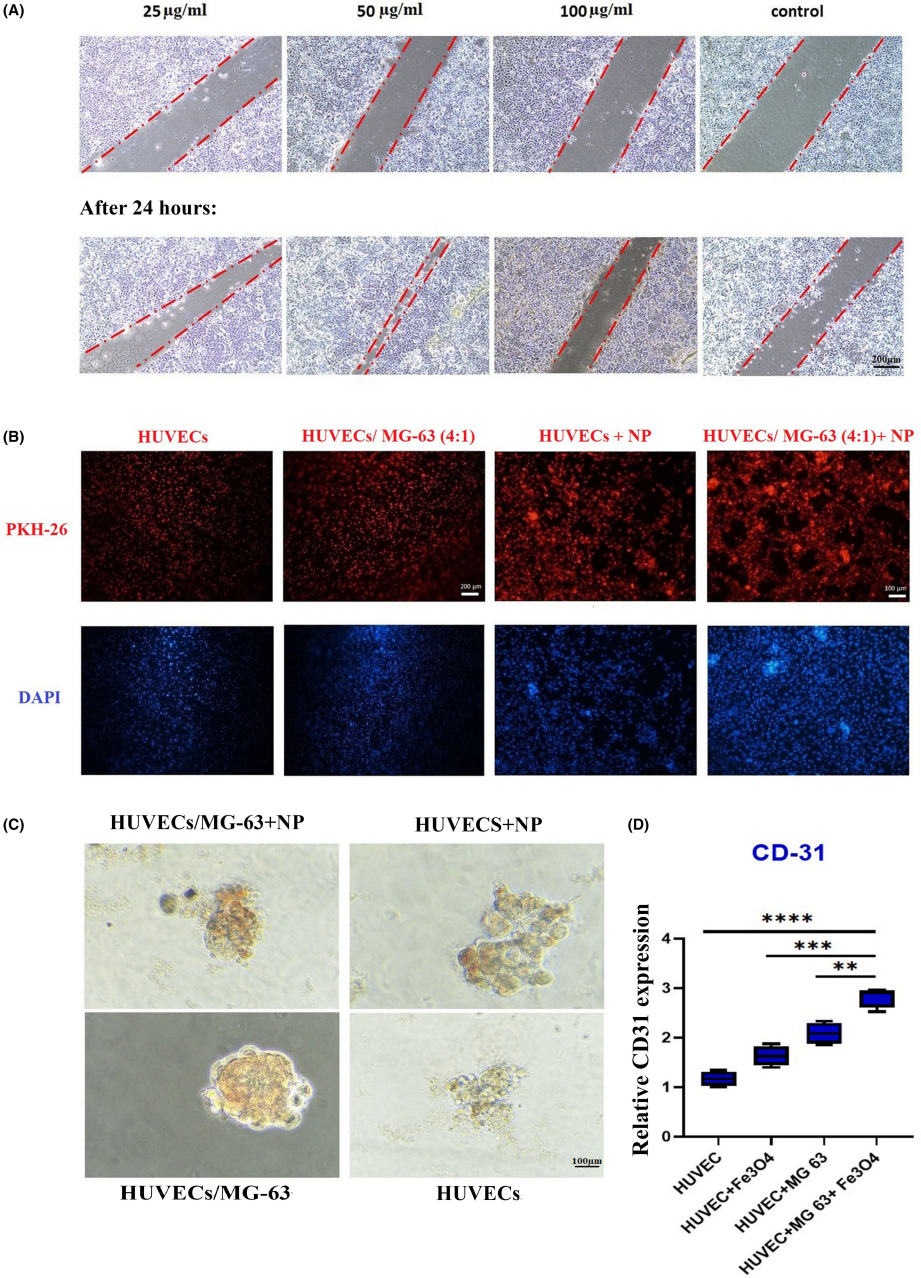
Tubular formation and angiogenesis development upon SPION administration; migration rate in scratched HUVECs/MG‐63s microtissues (scale bars: 100 μm) (A). Uptake of PKH26 fluorescent dye (red)‐labelled HUVECs and DAPI (Blue)‐stained nuclei in SPION‐free groups (scale bars: 200 μm) and SPION‐treated groups (scale bars: 100 μm) (B). Angiogenesis appearances in IHC test (C). CD‐31 gene expression levels (D) (*p < 0.05, **p < 0.01, ***p < 0.001).

The evaluation of cell migration using scratch and tube formation assays was conducted. Fifteen microgram per milliliter of SPION incubated with the scratched co‐culture of HUVECs‐MG63s at 4:1 significantly increased the migration rate compared to other doses administration. Furthermore, HUVECs co‐cultured with MG‐63s and 50 μg/mL of SPION could generate the highest cord‐like structures on a collagen feeder, all conducting to an effective tube formation (Figure 7).

### 3.7 SPION promoted angiogenesis

To investigate the angiogenesis, CD‐31 expression, as a marker demonstrating the presence of endothelial cells, was checked in both SPION‐treated and non‐treated groups, including group 1: HUVECs and group 2: HUVECs+ MG 63s (4:1). Based on the RT‐PCR results, the co‐culture of HUVEC and MG increases the expression of the angiogenic gene CD‐31 and has a significant difference with the SPION‐free HUVECs (*p* < 0.0001), HUVECs+ SPION (*p* = 0.002) and HUVEC+ MG‐63s (*p* = 0.0027) (Figure 7). In line with RT‐PCR results, SPION exposure in the IHC test resulted in the appearance of angiogenesis in the SPION‐treated groups (Figure 7).

### 3.8 Osteogenesis‐related gene expression levels

**FIGURE 8:**
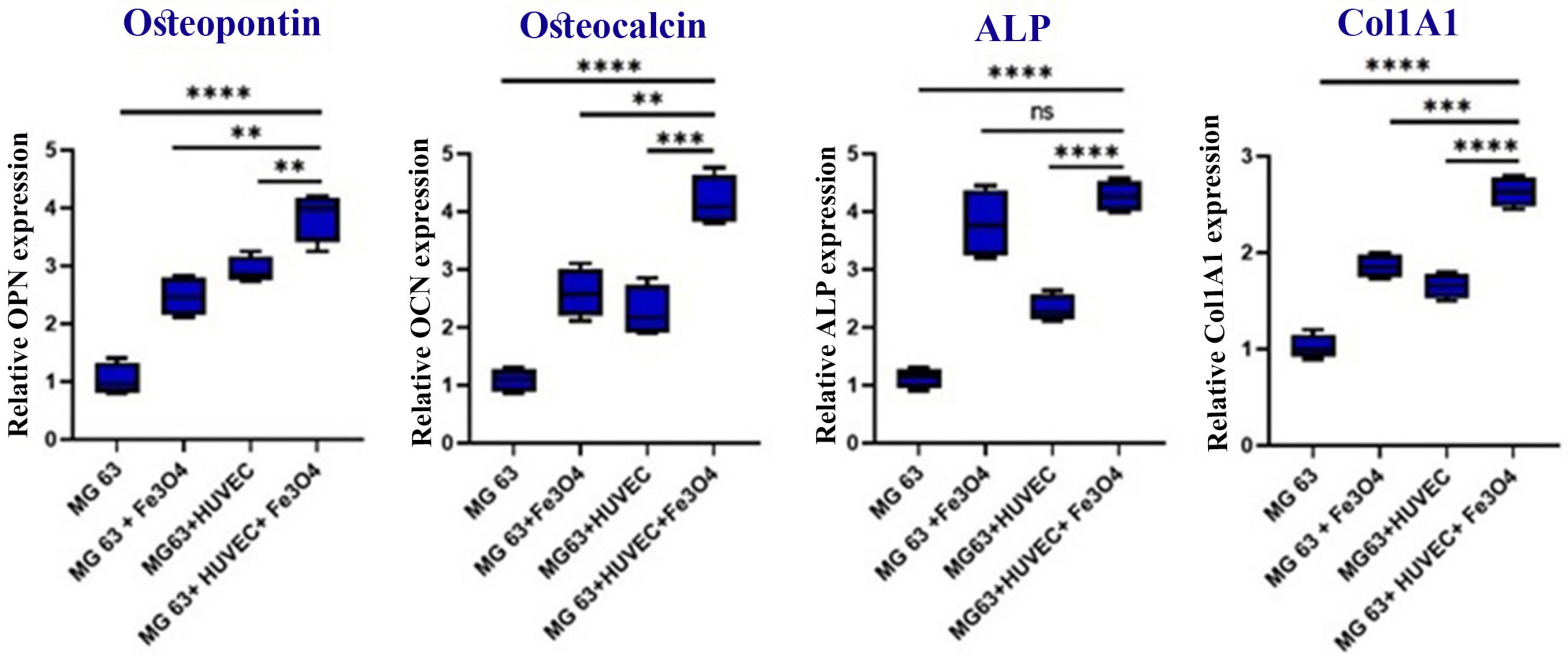
Osteogenesis‐related gene expression levels; gene expression levels of osteopontin (OPN), osteocalcin (OCN), ALP (Alkaline phosphatase) and collagen I (COL I) (*p < 0.05, **p < 0.01, ***p < 0.001, ****p < 0.0001).

The levels of expression of osteogenesis markers, such as ALP (an early osteogenic marker of bone formation and bone calcification), COL‐I (a collagenous protein in the bone matrix), and non‐collagenous proteins like osteocalcin (OCN) and OPN in the bone matrix, were assessed. The treated groups were compared to the non‐treated MG‐63 control group as the representation of bone tissue's main element. It is demonstrated that SPION significantly boosts the OPN expression in the SPION‐treated MG‐63 group (*p* = 0.0006). Although no significant difference is shown between MG‐63 plus HUVECs and MG‐63 plus SPION groups (*p* = 0.0701), the OPN generation level is highest in the MG‐63/HUVECs plus SPION group (*p* < 0.0001). In addition, OCN is illustrated to have a higher generation upon SPION application in the MG‐63 group (*p* = 0.0006). Likewise, HUVECs co‐culture showed a higher generation of this factor. There is no significant difference between the two later groups (*p* = 0.3315). Also, the MG‐63/HUVECs plus SPION group showed a particular increase in OCN expression compared to other groups (*p* < 0.0001). The ALP expression results show that although SPION and HUVEC both increase the expression of ALP in the MG‐63 group, the presence of SPION has a more significant effect than that of HUVEC (*p* = 0.0037). Also, the company of SPION and HUVEC next to MG‐63 compared to the MG group (*p* < 0.0001) and compared to the HUVEC+ MG group (*p* < 0.0001) increased ALP expression. The MG‐63 plus SPION and MG‐63 plus HUVECs groups had a higher expression of collagen type 1 than the control group, with no significant difference between the groups themselves (*p* = 0.552). Also, the synergistic effect of HUVEC+ MG‐63+ SPION significantly increased COL‐I expression compared to other groups. Overall, RT‐PCR results showed a combination of HUVEC+ MG‐63+ SPION leads to a synergic effect on the expression of osteogenesis markers (Figure 8).

## 4 DISCUSSION

While the bone tissue is generally capable of physiological regeneration and repair, managing substantial bone defects, particularly those resulting from tumours or infections, remains a significant challenge within the field of bone surgeries. These conditions may further lead to a severe delay in fusion or even non‐fusion, endangering patients' muscle function. Typically, bone grafts from the patient's body (autologous) or a donor (allogeneic) have been used to repair significant bone defects. Nevertheless, these grafts can be both time‐consuming and expensive to obtain. Therefore, a purpose‐built in‐vitro bone model similar to the natural tissue may represent an optimal alternative to natural grafts.^29^, ^30^

Moreover, 3D cell‐culture systems are greatly anticipated to be dependable in vitro tools to bridge the gap between animal studies and human trials in various investigations. This development has reached a stage where the United States Food and Drug Administration (FDA) announced that it no longer necessitates animal testing as a prerequisite for approving human drug trial conduction.^31^ This study aimed to develop an effective preclinical bone tissue structure involving osteoblasts and endothelial to mimic osteogenesis and angiogenesis features of natural bone tissue. This kind of model would be applied to comprehensive investigations and drug screening.

Based on the live‐dead, Alizarin Red reaction, ALP expression assays, tubular formation and IHC results in this investigation, 3D co‐cultured HUVECs: MG‐63s at a ratio of 4:1 results in the highest rate of viability, osteogenesis and angiogenesis. These data are supported by the fact that endothelial cells typically have a lower proliferation rate than osteoblasts. Due to this disparity, more HUVECs (endothelial cells) are needed to maintain a consistent interaction between osteoblast cells and endothelial cells within the co‐culture system. This ensures that the angiogenic and osteogenic processes are balanced and synchronized, promoting optimal cell communication and tissue development.^32^ This is consistent with a previous study by Unger et al., which reported the co‐culture of human dermal microvascular endothelial cells (HDMEC) and MG‐63s (HDMEC: MG‐63) in the ratios of 4:1 or 9:1 had sufficient quantities of both cell types after o1 week of cell culture.

In contrast, no endothelial cells were observed when the initial 1:1, 1:4 and 1:9 ratios were tested.^33^ Furthermore, as demonstrated in this study, the co‐culture of HUVECs and MG63s cells at the optimum ratio can facilitate cell interaction and the secretion of various factors that promote osteogenesis. This finding aligns with previous investigations that have shown a significant stimulation in endothelial growth factor (VEGF) and osteoblasts' differentiation and mineralization through the co‐immobilization of Human osteoprogenitors with HUVECs.^34^, ^35^

Additionally, MG‐63 cells can secrete bone morphogenetic proteins (BMPs), which are potent inducers of osteoblast differentiation and bone formation.^36^ BMPs can also induce the expression of VEGF in endothelial cells, which can further promote angiogenesis and osteogenesis.^37^ Furthermore, in line with the results of this experiment, Dariima et al. verified that endothelial cells and osteoblasts may support tubule‐like structures created by endothelial cells and that endothelial cells and osteoblasts synergistically boost the osteoblastic‐related gene expression by osteoblasts. It is also suggested that the process improves the mean tubule length in co‐culture models.^38^

To further investigate how to enhance the viability, osteogenic potential and angiogenic properties of the bone tissue models, different concentrations of SPIONs were introduced into the samples. The optimal effect of SPIONs was observed at 50 μg/mL. The toxicity of the SPIONs at a concentration of 100 μg/mL supports their dose‐dependent nature, which is consistent with the findings of a study by Naqvi et al. that indicates the toxicity of the higher concentrations of SPIONs to murine macrophage cells.^39^ Furthermore, applying 50 μg/mL of SPION resulted in significant expression of osteogenesis markers in RT‐PCR results. This was also indicated by calcium deposition observed through the Alizarin Red assay and ALP expression test. The increased activation of ALP following SPION administration is likely attributed to the de novo magnetic fields' properties, which trigger various cellular signalling pathways and facilitate their communication. This includes well‐known pathways such as the mitogen‐activated protein kinase^40^, ^41^, ^42^ and the BMP signal pathways.^43^, ^44^ This initially includes the over‐generation of RUNX2, a hallmark of early osteogenic differentiation, which plays a significant role in several key signalling pathways that support osteogenesis.^45^

**FIGURE 9:**
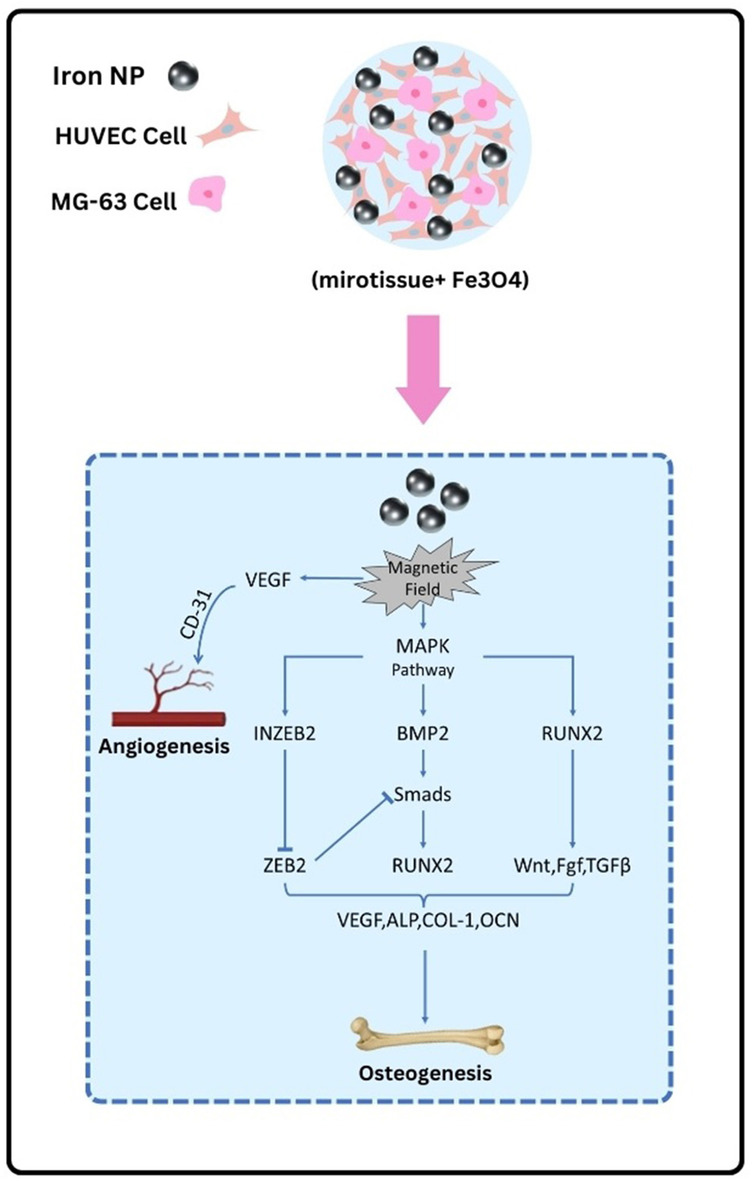
Schematic illustrating the signalling pathways involved in osteogenesis and angiogenesis.

On the other hand, BMP2 overexpression can activate Smads proteins, leading to more RUNX2 expression. These shape the BMP2/Smads/RUNX2 signalling pathway, which is essential for bone formation.^46^ Notably, SPION can up‐regulate INZEB2 levels, a crucial factor in maintaining osteogenesis because of its ability to down‐regulate the ZEB2 levels. ZEB2 is a marker that can inhibit the BMP2/Smads/RUNX2 signalling pathway.^47^ These mechanisms elucidate the effect of MNPs on osteogenesis by markedly stimulating transforming growth factor β and WNT factors (Figure 9).

These finally lead to up‐regulating the expression of ALP, collagen type I, OCN and growth factors that lead to cell growth, migration and, ultimately, osteogenesis.^43^, ^48^, ^49^, ^50^ Importantly, ALP plays a crucial role during the differentiation of osteoblasts. In addition, ALP is recognized as an early osteogenic marker involved in bone formation and calcification. Also, it is an enzyme that is secreted by osteoblasts, which are specialized bone‐forming cells. ALP plays a role in bone mineralization by providing a high phosphate concentration at the surface of the osteoblast cells. This phosphate is essential for depositing calcium and other minerals, facilitating the formation of hydroxyapatite, the main mineral component of bone. Therefore, ALP activity indicates osteoblast function and the early stages of bone mineralization.^51^, ^52^ Also, the experiments evaluating osteogenesis through the expression of bone protein matrix elements, such as OPN and OCN, demonstrated increased expression levels following exposure to SPIONs. This is supported by a study by Foroutan and Zaman Kasaei on the effect of SPION on osteoblast differentiation.^53^ In another study by Duan et al. SPION‐labelled human umbilical cord mesenchymal stem cells proved to be advantageous in osteonecrosis treatment by inhibiting apoptosis of bone cells through the Akt/Bcl‐2/Bad/Caspase‐3 signalling pathway.

Additionally, it enhanced bone regeneration by upregulating the osteogenic‐related proteins Osterix and Runx‐2.^54^ All these validate the unique physical, chemical and biological properties of SPIONs, making them an excellent option for biomedical applications in recent years. For instance, in a study on bone tissue regeneration, SPIONs have been shown to successfully increase the strength of the nanocomposite scaffold, promote calcium deposition and enhance ALP activity.^55^, ^56^

Their impact on angiogenesis further confirmed the efficacy of SPIONs. The results from CD‐31 expression in this study demonstrated a significant increase following the application of SPIONs. These findings agree with an in‐vivo survey where a gelatin sponge loaded with SPIONs showed increased bone mineral density and trabecular volume within the tissue. Notably, the histological examination revealed enhanced blood vessel formation concurrent with bone development. It was evident that osteoblasts and vascular endothelial cells had incorporated the SPIONs, resulting in heightened osteogenic and angiogenic capabilities.^19^ In another animal study by Singh et al. the development of new blood vessels alongside bone formation was observed when SPIONS were incorporated into polycaprolactone scaffolds.^57^

## 5 CONCLUSION

Taken together, the direct contact between osteoblast and endothelial cells significantly affects the bone model viability and osteogenesis. The optimum ratio has been suggested to be 4:1 (HUVEC: MG‐63). The samples' osteogenesis and angiogenesis further improved through 50 μg/mL SPION application, pointing out that it exerts its potential by up‐regulating the osteogenesis factors involving OPN, OCN, ALP and COL‐I as well as the angiogenesis factor CD‐31 that is warranted to be confirmed by in vivo studies.

Ultimately, the findings of this study suggest how SPION, as an optimum component in the context of the engendered microtissue in this study, can enhance the models to replicate the physiological nature of bone in terms of osteogenic activity and angiogenic potential. This can improve the interaction with surrounding tissues and help promote orthopaedic implants with superior performance, preventing device failure.

## AUTHOR CONTRIBUTIONS

**Maryam Dousti:** Data curation (equal); investigation (equal); project administration (equal). **Shima Parsa:** Investigation (equal); methodology (equal); writing – original draft (equal). **Farnaz Sani:** Formal analysis (equal); investigation (equal); methodology (equal). **Elham Bagherzadeh:** Investigation (equal). **Zahra Zamanzadeh:** Investigation (supporting). **Mahintaj Dara:** Investigation (equal). **Mahsa Sani:** Conceptualization (equal); data curation (equal); formal analysis (equal); funding acquisition (equal); project administration (equal); supervision (equal); writing – original draft (equal); writing – review and editing (equal). **Negar Azarpira:** Conceptualization (equal); data curation (equal); funding acquisition (equal); supervision (equal).

## FUNDING INFORMATION

This work was supported by a research deputy of Shiraz University of Medical Sciences (grant no. 30199).

## CONFLICT OF INTEREST STATEMENT

The authors declare that they have no conflicts of interest.

## CONSENT FOR PUBLICATION

Not applicable.

## Data Availability

All raw data from this study can be acquired by contacting the corresponding authors, Dr. Negar Azarpira and Mahsa Sani.
